# ClusterMap Building and Relocalization in Urban Environments for Unmanned Vehicles

**DOI:** 10.3390/s19194252

**Published:** 2019-09-30

**Authors:** Zhichen Pan, Haoyao Chen, Silin Li, Yunhui Liu

**Affiliations:** 1 School of Mechanical Engineering and Automation, Harbin Institute of Technology Shenzhen, Shenzhen 518055, China; zhchpan@163.com (Z.P.); lisilin013@163.com (S.L.); 2 Department of Mechanical and Automation Engineering, Chinese University of Hong Kong, Hong Kong, China; yhliu@mae.cuhk.edu.hk

**Keywords:** relocalization, SLAM, Localization, Map Descriptor, LIDAR-based Map Building, ClusterMap

## Abstract

Map building and map-based relocalization techniques are important for unmanned vehicles operating in urban environments. The existing approaches require expensive high-density laser range finders and suffer from relocalization problems in long-term applications. This study proposes a novel map format called the ClusterMap, on the basis of which an approach to achieving relocalization is developed. The ClusterMap is generated by segmenting the perceived point clouds into different point clusters and filtering out clusters belonging to dynamic objects. A location descriptor associated with each cluster is designed for differentiation. The relocalization in the global map is achieved by matching cluster descriptors between local and global maps. The solution does not require high-density point clouds and high-precision segmentation algorithms. In addition, it prevents the effects of environmental changes on illumination intensity, object appearance, and observation direction. A consistent ClusterMap without any scale problem is built by utilizing a 3D visual–LIDAR simultaneous localization and mapping solution by fusing LIDAR and visual information. Experiments on the KITTI dataset and our mobile vehicle illustrates the effectiveness of the proposed approach.

## 1. Introduction

Simultaneous localization and mapping (SLAM) supplies pose and map information for autonomous driving in unknown environments [1,2,3,4]. Precise localization ensures that vehicles run along pregenerated trajectories, and a good map provides a priori information about the surrounding environment that supports vehicles’ decision systems in predetermining driving commands. When an unmanned vehicle re-enters a familiar scene or loses its own location information, the vehicle should quickly regain its correct position in its operating environment to ensure safe driving. Position loss may be caused by a number of factors, such as temporary failure of sensors, rapid vehicle movement, and environmental changes. Practical unmanned vehicles should be able to relocalize independently by using existing maps and current environmental information.

In complex urban environments, 3D maps that reflect road conditions, obstacle locations, and other environmental information should be built to ensure safe driving. Existing autonomous driving solutions rely on high-precision and dense maps to realize vehicle navigation and even localization. Such maps provide accurate road/traffic descriptions and dense surrounding static information, and are thus a popular solution for commercial autonomous driving. Building these maps requires professional mapping devices and entails high building and maintenance costs. However, in many other scenarios, e.g., residential areas, factories, and campuses, high-precision maps are difficult to establish and maintain. 3D PointCloudMap is generally used as part of high-precision 3D maps to describe static objects in urban environments. The higher the point cloud densities, the more accurately the PointCloudMap reflects the details of environments. However, high densities lead to large map storage sizes and cause maps to contain a large amount of redundant information. These drawbacks increase the computational costs of localization and navigation approaches, which are based directly on PointCloudMap. A solution to the problem is OctoMap [5], which divides the whole 3D space into multiple cubes with a certain resolution. If a specific cube contains points belonging to PointCloudMap, then this cube is occupied by some obstacle. Relative to PointCloudMap, OctoMap can maintain a small data size and allow rapid processing. It is suitable for path planning and real-time obstacle avoidance.

To achieve a compact map representation and improve the usability of maps implemented in urban environments, we propose a new type of map called ClusterMap, on the basis of which a relocalization approach is developed. We first segment a point cloud into different point clusters according to the differentiation of objects without considering the category of each cluster, as illustrated in the work by the authors of [6]. Then, we design a cluster registration method to filter out the clusters belonging to dynamic objects to keep only the static clusters in the map. Finally, for each point cluster contained in the ClusterMap, we design a location descriptor by using the mutual positional relationship with its neighboring clusters. When an unmanned vehicle needs to complete a relocalization task, a local ClusterMap around the current vehicle’s location is built. Then, by matching location descriptors in local and global maps, the vehicle can establish a series of correspondences between two maps and, finally, obtain a relocalization result by calculating a transformation matrix on the basis of the correspondences. This relocalization method requires neither high-density point cloud data nor high-accuracy segmentation algorithms. Moreover, the developed location descriptor is strongly invariant to the changes of environmental objects’ appearance, illumination, and observation direction because it considers the spatial relationship among static environmental objects.

The paper is organized as follows. In Section 2, the current state of vision and LIDAR fusion-based SLAM and outdoor relocalization approaches is outlined. In Section 3.1, the SLAM method used for building ClusterMap is briefly introduced. In Section 3.2, the ClusterMap building algorithm and location descriptor are provided. In Section 4, we detail the relocalization algorithm, which is based on the ClusterMap. In Section 5, the experiments on the KITTI dataset and our experimental platform are provided to verify the proposed algorithms. Finally, in Section 6, the conclusions is given.

## 2. Related Work

Many SLAM approaches are available in the literature. Various sensors, such as the most common cameras and lasers, have also been used in map building and localization [7]. However, due to the complexity of outdoor scenes, a single type of sensor cannot be effective in all cases [8]. Mur-Artal et al. proposed an interesting framework called ORB-SLAM [9] using three threads to achieve tracking, local mapping, and loop closing. Other monocular SLAM approaches [10,11] still suffer from initialization and inconsistent scale issues. Although ORB-SLAM utilizes binocular and RGB-D cameras [12], it is limited by insufficient detection distances and high susceptibility to illumination changes. For 2D laser SLAM approaches, filter-based [13] and graph-based approaches [14] perform well in indoor structured scenes. However, in unstructured outdoor environments, the failure rates of 2D laser SLAM approaches increase because they are only able to use 2D laser range finders to perceive information in open and littered outdoor scenes.

Meanwhile, 3D SLAM approaches are more suitable for complex urban environments than 2D SLAM approaches. The 3D approaches in the literature [15,16,17] extract features such as lines, corners, and surfaces from point clouds to accelerate motion estimation. Some algorithms [18] are extended variants of 2D SLAM methods that use point cloud segments and leveled range scans to achieve 3D perceptions. Wang et al. [19] utilized 3D-LIDAR to precisely locate the autonomous vehicle, where the curbs information was detected to assist the pose estimation. Several experimental results were provided to demonstrate the accuracy and robustness of the method. The approaches proposed by Zhang et al. [20] and Zhang and Singh [2] enhance visual features by associating the depth information from LIDAR-based point clouds and obtain low-drift motion estimation results. Point clouds are downsized to maintain a constant point density. The downsized points are stored by using a 2D k-d tree. The depth values of visual features are obtained by finding and interpolating the three closest points of these visual features from the k-d tree. The fusion of visual and LIDAR information makes the motion estimation increasingly accurate because the method can use a series of 3D–3D or 3D–2D relations to recover the transformation matrix between two image frames.

Utilizing 3D point clouds for loop closure or relocalization is a challenging problem that has attracted increasing attention in the autonomous driving field. Lenac et al. [21] proposed a loop detection method that uses planar surface segments in point clouds as features in maps. The proposed method can achieve accurate and efficient SLAM in structured scenarios but not in unstructured environments. Visual features contain rich information and are distinguishable. As such, some approaches use visual information to assist loop detection. For example, Zhu et al. [22] and Chen et al. [23] used visual appearances to aid the loop closures in 3D SLAM. However, they used visual and LIDAR information separately without exploring the complementarity between sensors. Meanwhile, visual features with rich information also suffer from many limitations; for example, when the illumination or viewpoint changes are excessive, even robust features such as SIFT or SURF [24] can fail during matching. Other existing approaches [25,26] extract keypoints directly from 3D perceptions and construct relevant 3D Gestalt descriptors to describe each keypoint. Then, vote matrix voting from the nearest neighbors is used to find loops. SegMatch [27,28] provides a real-time algorithm for loop detection and localization on the basis of 3D point cloud segments. This method clusters and segments all the received point clouds and then calculates a corresponding descriptor for each segment. The k-d tree and machine learning approach are used for descriptor matching. Finally, a six-degree-of-freedom transformation matrix is obtained by geometric verification. However, the aforementioned approaches [25,26,27] require high-density point clouds for describing keypoints or segments. In addition, the feature descriptors are susceptible to environmental changes in long-term applications, in which shrubs or trees may change greatly, thereby causing a large change in the feature descriptors of the same object at different times. Furthermore, objects with high similarities in appearance, such as street lamps, vehicles, or other synthetic facilities, challenge the feature descriptor-based solutions.

Finman et al. presented an interesting work on object-based place recognition [29]. Their approach involves the use of a pregenerated primitive convolution kernel to convolute an entire point cloud and extract objects. It also constructs an object graph that represents the locational relationship among different objects. Through the matching of object graphs, places can be recognized despite appearance changes in a single object. However, the method can only handle small-scale scenarios, because the time to extract objects and the complexity of object graphs increase significantly for large-scale environments. Bogoslavskyi et al. [6] proposed a real-time object extraction solution with small computational requirements. The solution removes the ground from 3D scans and then clusters the point clouds into different clusters. However, the process is merely a pre-segmentation. Thus, the method cannot distinguish different clusters. Some descriptors, e.g., point feature histograms (PFHs) [30] and fast point feature histograms (FPFHs) [31], have been developed by encoding the neighborhood geometrical properties of points and using the average curvature of multidimensional histograms around points. PFHs and FPFHs can provide informative signatures for the feature representation of 3D points. They are also invariant to 6D movement and cope well even with large noises. However, PFHs and FPFHs are only suitable for high-precision 3D reconstruction, and are thus not valid for practical outdoor applications because high-density point clouds in outdoor environments are difficult to provide.

In view of the above problems, the present study aims to develop a novel type of map, i.e., the ClusterMap, which is more compact than the OctoMap and PointCloudMap. The proposed map contains point cluster information only, and thus it does not require a large storage space. This characteristic favors storage and transfer processes in many cloud-based applications. A cluster descriptor is also developed to distinguish different clusters in maps. It is used for map matching to realize relocalization. Relative to existing approaches, the ClusterMap-based relocalization method performs well even with low-density laser range finders, e.g., VLP-16. In addition, the algorithm is strongly robust to changes in environmental objects’ appearance, illumination, and observation direction. The ClusterMap-based relocalization method can be also used for loop closure, which we will tackle in our future work.

## 3. ClusterMap Building

In this section, the process of building the ClusterMap and the details of the descriptor for each cluster in the map are presented. The ClusterMap and the cluster descriptor are used to achieve the relocalization of unmanned vehicles in urban environments.

### 3.1. SLAM for ClusterMap Building

Generally, the basic requirement for map building is precise localization information. To enhance the consistency of the ClusterMap with the real world, we need to obtain accurate location information in urban environments. The SLAM can be treated as a black box that provides consistent pose estimation, and this estimation is very important to build consistent ClusterMap. Because SLAM research is not the focus of the paper, we implement SLAM by directly integrate the visual–LIDAR odometry [20] into the framework of ORB-SLAM [9,12]. The visual–LIDAR odometry fuses the sensor data received from a monocular camera and a VLP-16 LIDAR, to extract depth information for 2D visual features by using the advantages of different sensors. The ORB-SLAM implements loop closures and uses global nonlinear optimization algorithms [32] to adjust odometry and map features synchronously. The loop closures eliminate the accumulated errors generated during motion estimation. The ORB-SLAM integrated with visual–LIDAR odometry provides good pose references for constructing the ClusterMap. By considering the self-consistency of the work, the SLAM framework is introduced briefly. For the details, please refer to the references herein [12,20].

**Figure 1:**
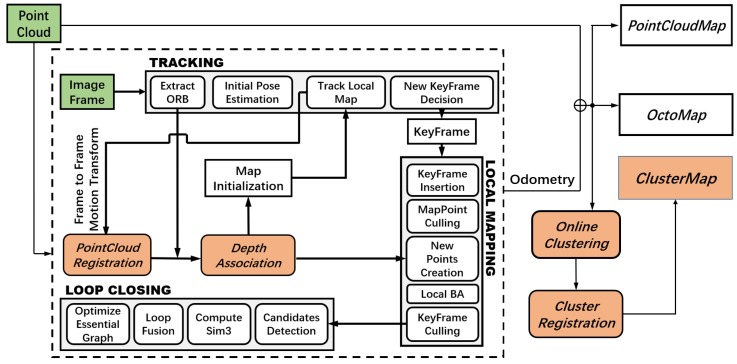
Pipeline of the mapping framework including the simultaneous localization and mapping (SLAM) and ClusterMap building.

Figure 1 shows the pipeline of the mapping framework. The 3D SLAM is indicated by a dashed box in Figure 1. It includes the threads of TRACKING, LOCAL MAPPING, and LOOP CLOSING [12], which are further expanded with two blocks, i.e., PointCloud Registration and Depth Association [20]. The localization results from SLAM are continuously utilized to register point clouds and generate maps such as PointCloudMap, OctoMap, or ClusterMap.

The transformation between image frames is calculated in the TRACKING thread, as shown in Figure 1, according to the 2D–3D relations between 2D visual features and 3D local map points. With the localization result in the TRACKING thread, several recent frames of LIDAR data are accumulated in the point cloud registration to increase the density of the perceived point clouds. The process enables the usage of low-density LIDAR, e.g., VLP-16, to achieve a performance similar to that of HDL-64 or other dense LIDARs, where the point cloud is reprojected to the synchronized image by using the extrinsic calibration result between a camera and a LIDAR [33]. The point cloud is stored by using a 2D k-d tree constructed in accordance with the coordinates of each point. Meanwhile, to retain as much structural information as possible, the k-d tree is searched for all 3D points within a range from a visual feature $f_{i}$ in the camera frame. Let ${\{}^{i}P\}=\left\{{}^{i}P_{1},{}^{i}P_{2},\ldots ,{}^{i}P_{n}\right\}$ denote the set of neighboring 3D points, where ${}^{i}P_{j}={\left[x_{j},y_{j},z_{j}\right]}^{T}$ represents the 3D coordinates of the *j*th point. Let $f_{i}=z_{i}\;{\left[x_{i},y_{i},1\right]}^{T}$ denote the coordinates of the depth-enhanced visual feature, where $z_{i}$ is the unknown depth parameter. Then, the three points with the smallest distance are selected from ${\{}^{i}P\}$. The three points form a local planar patch in the 3D space. The visual feature $f_{i}$ is treated as a point on the patch. The depth parameter $z_{i}$ is obtained by solving the following equation,

(1) $${\left(f_{i}-{}^{i}P_{n_{1}}\right)}^{T}\left(\left({}^{i}P_{n_{1}}-{}^{i}P_{n_{2}}\right)\times \left({}^{i}P_{n_{1}}-{}^{i}P_{n_{3}}\right)\right)=0,$$

where ${}^{i}P_{n_{1}},{}^{i}P_{n_{2}},$ and ${}^{i}P_{n_{3}}$ are the three selected points with known 3D information. When detected visual features are initialized by calculating the depth parameter, they are directly registered in the map with low-scale deviation. The initialization of monocular odometry is therefore simplified to benefit the building of a consistent map. Using the above method, a significant portion of the features (~35–75% in our experiments) in a keyframe can be associated with depth information; keyframes are then selected and processed in the LOCAL MAPPING thread [12]. Compared with the triangulation method that only uses the same visual features observed in multiple image frames to estimate depth values, the depth association developed in the work by the authors of [20] exhibits enhanced computation efficiency. It also makes SLAM further accurate and robust because the triangulation method depends on visual odometry initialization, which suffers from scale and data association problems.

The above SLAM method is used to provide location info for building the ClusterMap, and the ClusterMap is then used for relocalization. The relocalization problem is a key technology in many robotic navigation applications of unmanned vehicles. For long-term applications, visual appearance-based approaches are inefficient due to the significant changes in illumination or objects in environments, as discussed in Section 1 and Section 2. In addition, unmanned vehicles driving in urban environments often face a situation in which they enter the same place, but from different directions. The appearances of the same place observed from different directions are significantly different. This characteristic leads to difficulties in relocalization. Furthermore, the existing LIDAR-based relocalization approaches all depend on dense point clouds. In the present study, a novel map named the ClusterMap and relocalization algorithms are developed for long-term unmanned vehicle applications.

### 3.2. Building ClusterMap

From the SLAM, accurate and scale consistent trajectories of unmanned vehicles can be obtained. In the block of Online Clustering in Figure 1, the method published by Bogoslavskyi and Stachniss [6] is utilized to segment the point cloud received from the current LIDAR frame into different clusters. However, the clustering method [6] only produces clustering results in consequent frames without providing associations for clusters belonging to the same object. Therefore, in the cluster registration block in Figure 1, each cluster is associated with corresponding odometry information; then, all clusters are registered into the same map frame. Assuming that dynamic objects do not appear frequently in the same place, clusters derived from dynamic objects are removed by judging whether clusters are appearing in the same location. Furthermore, the method by Bogoslavskyi and Stachniss [6] cannot always guarantee the consistency of clustering; for example, in some cases, only the trunk of a tree is segmented, whereas in other cases, only the canopy is segmented. To address this problem, Algorithm 1 is developed, which can piece together multiple clusters that belong to different parts of the same object. Let $\left\{C\right\}=\left\{C_{1},C_{2},\ldots ,C_{m}\right\}$ be the set of registered clusters. $\forall C_{i}\in \left\{C\right\},\;C_{i}=\left\{\left\{P_{i}\right\},O_{i},ap_{i}\right\}$, where $\left\{P_{i}\right\}=\left\{p_{0},p_{1},\ldots ,p_{n}\right\}$ is the set of points belonging to $C_{i}$, where $O_{i}=\left[\;\left(\sum p_{n}\right)/n\;\right]$ is the virtual center of $C_{i}$ and $ap_{i}$ is the cluster’s occurrence number. Let $C_{x}=\left\{\left\{P_{x}\right\},O_{x},ap_{x}\right\}$ denote the cluster to be registered. In Algorithm 1, the function *sqrDist()* on lines 2 and 5 is used to calculate the squared distance between two clusters, in accordance with virtual center points; function *radiusSearch()* on line 10 returns the number of points belonging to $\left\{P_{i}\right\}$ and within the range of a sphere, with $p_{j}$ as the center and *rad* as the radius.

**Algorithm 1** Cluster Registration.**Require:**{C}:Set of registered clusters **Require:**$C_{x}$:Cluster waiting for registration **Require:**$C_{0},C_{1},C_{2}\in \left\{C\right\}$:Three clusters closest to $C_{x}$ in {C} 1: **for** each i∈{0,1,2}
**do** 2: **if**
*sqrDist*($O_{i},O_{x}$)>*maxDist*
**then** 3:  $\left\{C\right\}\leftarrow \left(C_{x}\;\&\;ap_{x}=1\right)$; break; 4: **end if** 5: **if**
*sqrDist*($O_{i},O_{x}$)<*minDist*
**then** 6:  $\left\{P_{i}\right\}\leftarrow \left(\left\{P_{x}\right\}\;\&\;ap_{i}++\right)$; 7: **else**
 8:  count←0; 9:  **for all**
$p_{j}\in \left\{P_{x}\right\}$
**do** 10:   **if**
*radiusSearch*($p_{j}$,$\left\{P_{i}\right\}$,*rad*)>*minNum*
**then** 11:    count++; 12:   **end if** 13:  **end for** 14:  **if**
count>*sizeof*($\left\{P_{x}\right\}$) / *thresholdNum*
**then** 15:   $\left\{P_{i}\right\}\leftarrow \left(\left\{P_{x}\right\}\;\&\;ap_{i}++\right)$; 16:  **end if** 17: **end if** 18: **end for**

**Figure 2:**
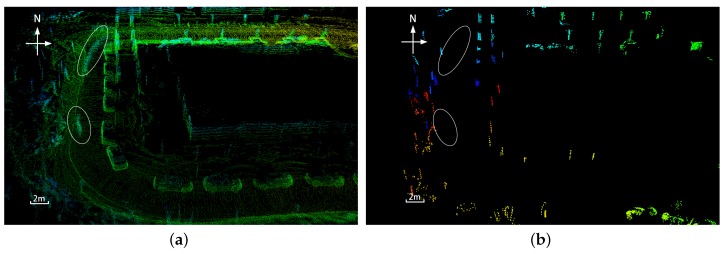
Demonstration of PointCloudMap and ClusterMap built from the same dataset. (**a**) PointCloudMap contains trailing smears caused by dynamic pedestrians; (**b**) ClusterMap reserves only static objects.

The registration algorithm retains the clusters with $ap_{i}$ greater than a predefined threshold. For example, if $ap_{i}>10$, then only clusters that appear more than 10 times in the same location are considered to be generated by a common static object. Figure 2 illustrates PointCloudMap in panel a and the ClusterMap in panel b created from the same dataset. The PointCloudMap is built by continuously attaching the 3D point cloud data along the trajectory poses estimated by SLAM. As shown in Figure 2a, moving pedestrians leave trailing smears, marked as circles in the map. Meanwhile, a large number of redundant points are filtered out in the ClusterMap, and only point clusters belonging to specific objects, e.g., trees, shrubs, street lamps, and wall columns, are retained. As shown in Figure 2b, the moving objects are removed. Compared with PointCloudMap, the ClusterMap is more compact, and it reserves almost all dominant static objects; thus, it can be processed much faster.

### 3.3. Cluster Descriptor for Clusters in ClusterMap

**Figure 3:**
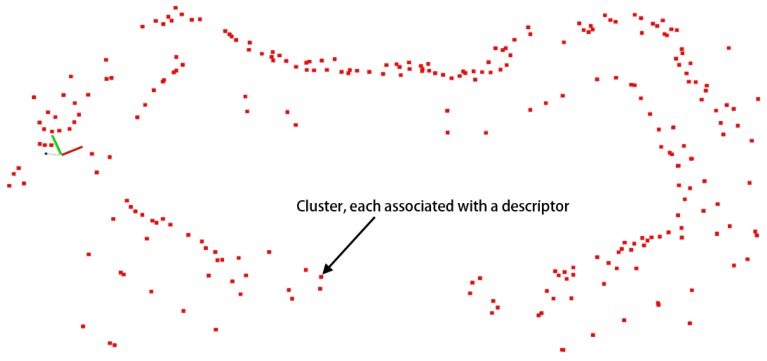
Building of 2D ClusterMap by projecting all clusters to a 2D horizontal plane.

Similar with the feature descriptors in the field of computer vision [12], cluster descriptors are developed to distinguish different clusters in the ClusterMap. The cluster matching between the global and local ClusterMap is then achieved according to the descriptor. The descriptor is defined by describing the mutual spatial relationship among different objects. Generally, environmental objects are roughly distributed on a ground plane in many outdoor applications, and the height values of different objects are not clearly different. The main purpose of relocalization is to determine the location of a vehicle in a global map quickly. As such, considering the height difference of environmental objects is not entirely significant. Therefore, the 3D clusters perceived in the previous sub-section are projected on the 2D plane for simplicity, as shown in Figure 3. The cluster descriptor is created for the simplified 2D clusters. The cluster descriptor can also be extended to 3D ClusterMap, which will be studied in our future work. We project the 3D ClusterMap to the 2D plane by setting the third value of $O_{i}$ equal to zero. The 2D ClusterMap is used for relocalization in Section 4.

**Figure 4:**
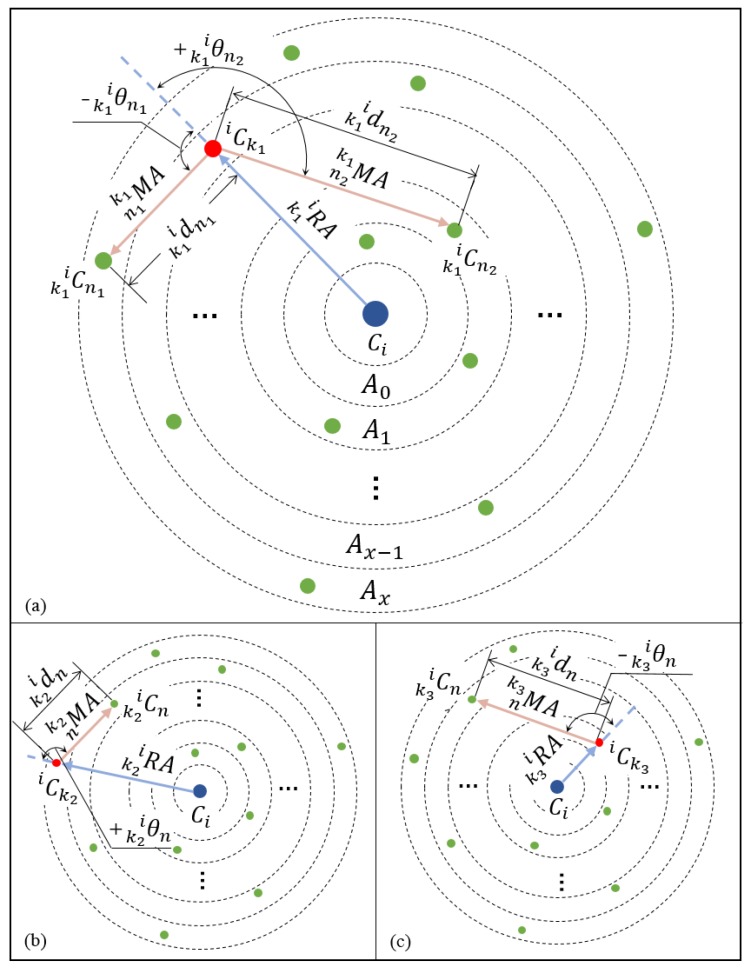
Process of constructing subdescriptors of $C_{i}$. (**a**–**c**) Illustrations of different neighboring clusters of $C_{i}$.

Let us denote $L_{i}$ as the descriptor associated with cluster $C_{i}\in \left\{C\right\}$, and SR as a neighboring radius of clusters. Note that $L_{i}$ is a third-order tensor that stores the mutual spatial relationship among different objects in the range of SR. The larger the SR, the more detailed the description for a cluster; however, the time needed to establish the descriptor and match clusters also increases. Let ${\{}^{i}C\}$ denote the set of neighboring clusters of $C_{i}$, and let

$${\{}^{i}C\}={\{}^{i}C_{1}{,}^{i}C_{2},\ldots {,}^{i}C_{n_{nb}}\},$$

where $n_{nb}$ denotes the number of neighboring clusters in ${}^{i}C$. Then, as illustrated in Figure 4a, a series of concentric circles are used to divide ${\{}^{i}C\}$ into X parts with equal annulus widths. Each annulus contains an unequal number of clusters belonging to ${\{}^{i}C\}$. The green dots denote the clusters. For $\forall {}^{i}C_{k}\in {\{}^{i}C\}$, a subdescriptor is built, denoted as ${}^{i}L_{k}$. It is part of the entire descriptor for $C_{i}$. To build the subdescriptor of ${}^{i}C_{k}$, we first define a reference axis ${}_{k}^{i}\vec{RA}$ from $C_{i}$ to ${}^{i}C_{k}$ as

$$\begin{array}{cc} {}_{k}^{i}\vec{RA} & =\vec{C_{i}{}^{i}C_{k}}, \end{array}$$

where $\vec{C_{i}{}^{i}C_{k}}$ denotes the vector from $C_{i}$ to ${}^{i}C_{k}$. Then, a measurement axis ${}_{n}^{k}\vec{MA}$ from ${}^{i}C_{k}$ to ${}_{k}^{i}C_{n}\in {\{}_{k}^{i}C\}$ is defined as

$$\begin{array}{cc} {}_{n}^{k}\vec{MA} & =\vec{{}^{i}C_{k}{}_{k}^{i}C_{n}}, \end{array}$$

where $\vec{{}^{i}C_{k}{}_{k}^{i}C_{n}}$ denotes the vector from ${}^{i}C_{k}$ to ${}_{k}^{i}C_{n}$, and

(2) $${\{}_{k}^{i}C\}:={\{}_{k}^{i}C_{n}={}^{i}C_{n}\in {\{}^{i}C\}|n\neq k\}.$$

Figure 4a–c demonstrates the process of constructing subdescriptors for different neighboring clusters. On the basis of ${}_{k}^{i}\vec{RA}$ and ${}_{n}^{k}\vec{MA}$, as shown in Figure 4a, a parameter vector ${}_{k}^{i}L_{n}=\left[{\;}_{k}^{i}d_{n}\;,{\;}_{k}^{i}\theta_{n}\;\right]$ is used to define the locational relationship between ${}^{i}C_{k}$ and ${}_{k}^{i}C_{n}$ as

(3) $$\begin{array}{cc} {}_{k}^{i}d_{n} & {=∥}_{n}^{k}\vec{MA}∥\\ {|}_{k}^{i}\theta_{n}| & =acos(\frac{{}_{k}^{i}\vec{RA}\cdot_{n}^{k}\vec{MA}}{{∥}_{k}^{i}\vec{RA}{∥∥}_{n}^{k}\vec{MA}∥}), \end{array}$$

where ${|}_{k}^{i}\theta_{n}|$ is the angle from ${}_{n}^{k}\vec{MA}$ to ${}_{k}^{i}\vec{RA}$ obeying the right-hand rule. The sign of ${}_{k}^{i}\theta_{n}$ is consistent with the z-value of ${}_{k}^{i}{\vec{n}}_{n}$, where

(4) $${}_{k}^{i}{\vec{n}}_{n}={}_{n}^{k}\vec{MA}\times {}_{k}^{i}\vec{RA}.$$

By stacking the above parameter vectors for members in ${\{}_{k}^{i}C\}$, defined in (2), we obtain the following subdescriptor of ${}^{i}C_{k}$,

(5) $${}^{i}L_{k}=\left[{\;}_{k}^{i}L_{1},{\;}_{k}^{i}L_{2},\ldots ,{\;}_{k}^{i}L_{n_{nbe}}\;\right],$$

where $n_{nbe}=n_{nb}-1$ denotes the number of elements in ${\{}_{k}^{i}C\}$. Each element in ${}^{i}L_{k}$ is calculated with (3). Note that each neighboring cluster of a cluster is associated with a subdescriptor. And, by stacking all these subdescriptors, we obtain the complete cluster descriptor $L_{i}$ of $C_{i}$, which is given as

$$L_{i}=\left[{\;}^{i}L_{1},{\;}^{i}L_{2},\ldots ,{\;}^{i}L_{n_{nb}}\;\right].$$

At the same time, $n_{nb}$ members exist in ${\{}^{i}C\}$, and $n_{nbe}$ members exist in ${\{}_{k}^{i}C\}$. Therefore, the computation complexity of building the entire cluster descriptor $L_{i}$ is $O(n_{nbe}*n_{nb})$.

To accelerate the descriptor matching process, we divide the subdescriptors into different parts in accordance with their location annulus, as shown in Figure 4. The series of annulus are labeled as $A_{j},j\in 1,\ldots ,n_{a}$, where $n_{a}$ denotes the number of annuli. The annulus’s label $A_{j}$ is used to mark the subdescriptors in different parts. Then, the entire descriptor of $C_{i}$ is given as

$$\begin{array}{cc} L_{i}=[\; & {\left[{\;}^{i}L_{k_{11}},\ldots {,}^{i}L_{k_{1n_{A1}}}\;\right]}^{A_{1}},{\left[{\;}^{i}L_{k_{21}},\ldots {,}^{i}L_{k_{2n_{A2}}}\;\right]}^{A_{2}},\ldots \;,\\ \; & {\left[{\;}^{i}L_{k_{n_{a}1}},\ldots {,}^{i}L_{k_{n_{a}n_{An_{a}}}}\;\right]}^{A_{n_{a}}}\;]. \end{array}$$

**Figure 5:**
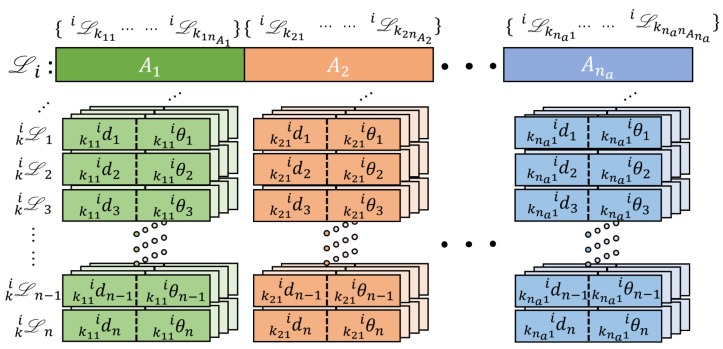
Third-order tensor structure of location descriptor $L_{i}$, which is divided into X parts. $A_{j}$ indexes the *j*-th annulus shown in Figure 4

The structure of $L_{i}$ illustrated in Figure 5 is stored by using a third-order tensor. The numbers $n_{A_{j}},j\in \left(1,\ldots ,n_{a}\right)$ are not the same. As shown in Figure 5, each subdescriptor ${}^{i}L_{k}$ is represented using a page that contains *n* rows, where each row represents ${}_{k}^{i}L_{n}$ defined in (3), consisting of a distance (d) and an angle (θ) relative to ${}^{i}C_{k}$ and ${}_{k}^{i}C_{n}$. For example, if there are $n_{A_{j}}$ clusters included in $A_{j}$, then the $A_{j}$ area in Figure 5 contains $n_{A_{j}}$ pages, each representing a subdescriptor ${}^{i}L_{k}$.

## 4. Relocalization Algorithm Based on ClusterMap

To relocalize a vehicle, we build a local ClusterMap of the surrounding environment at the vehicle’s current location. The descriptor for each cluster in the map is established by using the proposed method in Section 3.3. By performing descriptor matching between clusters in the local ClusterMap and a prebuilt global ClusterMap {C}, a series of cluster correspondences between the two maps is obtained. The correspondences may contain wrong matches. Therefore, three geometric conditions are utilized to remove the outliers. Finally, the relocalization is realized by calculating the transformation between the local and global maps in accordance with the obtained cluster correspondences.

### 4.1. Cluster Descriptor Matching

The variable notations for a locally built ClusterMap are associated with a hat, e.g., the set of all clusters ${\hat{C}}_{j}$ included in the local map is $\left\{\hat{C}\right\}$. Each cluster is followed by a descriptor ${\hat{L}}_{j}$, and so on. The clusters from local and global ClusterMap are derived from the environmental objects. As such, two clusters that belong to the same object should have at least similar descriptors—the two clusters match each other. As discussed previously, the transformation between maps can be calculated on the basis of the matches. To measure the similarity between two clusters $C_{i}$ and ${\hat{C}}_{j}$, the distance $D_{ji}$ between descriptors $L_{i}$ and ${\hat{L}}_{j}$ is defined. First, for each subdescriptor ${}^{j}{\hat{L}}_{k_{1}}\in {\hat{L}}_{j}$ with the annulus label of $A_{x}$, the subdescriptors in $L_{i}$ with the same annulus label are searched to find matches; the matching error between two subdescriptors is denoted as ${}^{ji}D_{k_{1}k_{2}}$. $D_{ji}$ is proportional to the sum of all subdescriptor matching errors; the smaller $D_{ji}$, the smaller distance between $C_{i}$ and ${\hat{C}}_{j}$.

**Figure 6:**
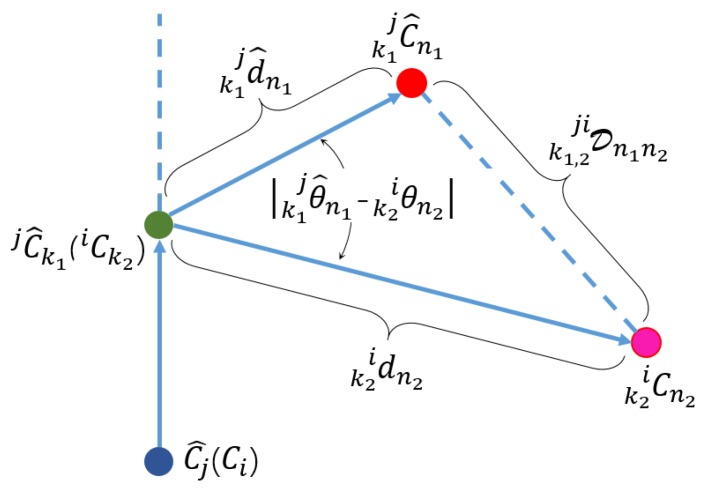
Geometric distance between two parameter vectors.

However, considering the matching error only can cause some problems. For example, in matching ${\hat{C}}_{1}$ with $C_{2}$ and $C_{3}$, ${\hat{L}}_{1}$ has 10 successful subdescriptor matches with $L_{2}$, with each matching error being about 1.0m, whereas ${\hat{L}}_{1}$ has only one successful match with $L_{3}$, where its matching error being 0.1m. If the matching error is unique judging criterion, then $C_{3}$ becomes the best match for ${\hat{C}}_{1}$. However, $C_{2}$ should be the better match for ${\hat{C}}_{1}$ because ${\hat{C}}_{1}$ and $C_{2}$ are likely derived from the same environmental object on the basis of the number of successful subdescriptor matches between them. Therefore, matching error and matching number are considered simultaneously. The distance $D_{ji}$ between $L_{i}$ and ${\hat{L}}_{j}$ is obtained as

(6) $$\begin{array}{c} D_{ji}=\left(\frac{1}{n_{suc}}-\frac{1}{\hat{n_{nb}}}\right)\sum_{k_{1},k_{2}\in S_{suc}}{{}^{ji}D}_{k_{1}k2},\forall j\in \left[1,N_{l}\right],i\in \left[1,N_{g}\right], \end{array}$$

where $n_{suc}$ is the size of successful match set $S_{suc}$, $\hat{n_{nb}}$ is the size of ${}^{j}\hat{C}$, and $N_{l}$ and $N_{g}$ denote the total number of clusters included in $\left\{\hat{C}\right\}$ and {C}, respectively. A key point in this study is to determine whether two subdescriptors are successfully matched. As shown in Figure 6, a geometric distance ${}_{k_{1,2}}^{ji}D_{n_{1}n_{2}}$ between ${}_{k_{1}}^{j}{\hat{L}}_{n_{1}}$ and ${}_{k_{2}}^{i}L_{n_{2}}$ is defined as

(7) $$\begin{array}{c} \begin{array}{cc} {}_{k_{1,2}}^{ji}D{\;}_{n_{1}n_{2}}^{2} & ={}_{k_{1}}^{j}{\hat{d}}_{n_{1}}^{2}+{}_{k_{2}}^{i}d_{n_{2}}^{2}\\ \; & -2\cdot {}_{k_{1}}^{j}{\hat{d}}_{n_{1}}\cdot {}_{k_{2}}^{i}d_{n_{2}}\cdot cos(|{}_{k_{1}}^{j}{\hat{\theta }}_{n_{1}}-{}_{k_{2}}^{i}\theta_{n_{2}}\left|\right), \end{array} \end{array}$$

where ${}_{k}^{i}L_{n}=\left({\;}_{k}^{i}d_{n}\;,{\;}_{k}^{i}\theta_{n}\;\right)$ is defined in (3). Then, the matching error ${}^{ji}D_{k_{1}k_{2}}$ between two subdescriptors ${}^{j}{\hat{L}}_{k_{1}}$ and ${}^{i}L_{k_{2}}$ is calculated as

(8) $${}^{ji}D_{k_{1}k_{2}}=\left(\frac{1}{n_{suc,k_{1,2}}}-\frac{1}{{\hat{n}}_{nbe,k_{1}}}\right)\sum_{n_{1},n_{2}\in S_{suc,k_{1,2}}}{}_{k_{1,2}}^{ji}D_{n_{1}n_{2}}\;\;\;\;$$

where $n_{suc,k_{1,2}}$ is the size of the set $S_{suc,k_{1,2}}$, with the geometric distances defined in (7) being less than a predefined threshold; ${\hat{n}}_{nbe,k_{1}}$ is the size of ${}_{k_{1}}^{j}\hat{C}$. If the matching error ${}^{ji}D_{k_{1}k_{2}}$ is less than a predefined threshold $T^{\prime }$, then ${}^{j}{\hat{L}}_{k_{1}}$ and ${}^{i}L_{k_{2}}$ match successfully with each other and are used to calculate $D_{ji}$ in (6). If $D_{ji}$ is less than the threshold *T*, then $C_{i}$ is added to a set ${\{}^{j}\hat{C}\}$ containing all clusters matched to $\hat{C_{j}}$, which is defined as

(9) $${\{}^{j}\hat{C}\}={\{}^{j}{\hat{C}}_{i_{1}}{,}^{j}{\hat{C}}_{i_{2}},\ldots {,}^{j}{\hat{C}}_{i_{ca}}\},$$

where $i_{ca}$ denotes the size of ${\{}^{j}\hat{C}\}$. The more distinguishable ${\hat{L}}_{j}$ is, the fewer candidates are, i.e., the smaller $i_{ca}$ is. Furthermore, the threshold *T* is an empirical value roughly set to 1.5∼2.0 times the accuracy of the ClusterMap, e.g., T=0.06 m in our experiments. Although the entire {C} is queried for each member in $\left\{\hat{C}\right\}$, the time consumption of descriptor matching is within an acceptable range owing to the small size of $\left\{\hat{C}\right\}$, usually between 10 and 25. A computation analysis is further provided in the experimental section.

### 4.2. Removing Outliers Based on Geometric Verification

**Figure 7:**
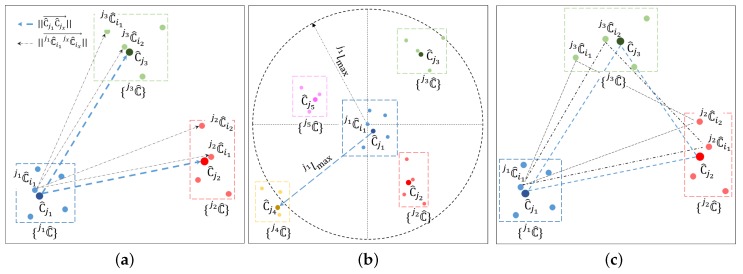
Demonstration of the three geometric conditions, where different candidate sets are indicated by different colors: (**a**) length condition, (**b**) inclusion condition, and (**c**) triangular condition.

After acquiring the sets of ${\{}^{j}\hat{C}\},j\in \left(1,\ldots ,N_{l}\right)$, several geometric verification rules are then defined to filter out match outliers. If a cluster ${}^{j_{1}}{\hat{C}}_{i_{1}}\in {\{}^{j_{1}}\hat{C}\}$ and ${\hat{C}}_{j_{1}}$ matches correctly, then they should be generated with the same object. Thus, ${}^{j_{1}}{\hat{C}}_{i_{1}}$ satisfies the following three geometric conditions, as illustrated in Figure 7.

- Length condition: Use distances between clusters included in $\left\{\hat{C}\right\}$ to filter out some unsatisfied candidates. In any other set, ${\{}^{j_{x}}\hat{C}\}$, a cluster, ${}^{j_{x}}{\hat{C}}_{i_{x}}$, should be found so that
  (10) $$(∥\vec{{}^{j_{1}}{\hat{C}}_{i_{1}}{\;}^{j_{x}}{\hat{C}}_{i_{x}}}∥-∥\vec{{\hat{C}}_{j_{1}}\;{\hat{C}}_{j_{x}}}{∥)}^{2}<{T\;}^{2},$$
  where *T* is the same as the threshold mentioned above.
- Inclusion condition: Let ${}^{j_{1}}l_{max}$ be the maximum distance between ${\hat{C}}_{j_{1}}$ and all other clusters in the local ClusterMap $\left\{\hat{C}\right\}$. Therefore, $N_{l}$ clusters are present in the circle, with ${\hat{C}}_{j_{1}}$ as the center and ${}^{j_{1}}l_{max}$ as the radius. Correspondingly, in the global ClusterMap, ~$N_{l}$ clusters are available in the circle, with ${}^{j_{1}}{\hat{C}}_{i_{1}}$ as the center and ${}^{j_{1}}l_{max}$ as the radius. The cluster ${}^{j_{1}}{\hat{C}}_{i_{1}}$ is preserved only if enough different groups exist in this circular range.
- Triangular condition: A cluster ${\hat{C}}_{j_{1}}$ and every two other clusters in $\left\{\hat{C}\right\}$ can form a base triangle (the blue dotted triangle shown in Figure 7c); if clusters in the corresponding groups can form a triangle similar to the base one, then the cluster ${}^{j_{1}}{\hat{C}}_{i_{1}}$ is retained. By randomly selecting two clusters from $\left\{\hat{C}\right\}$ except ${\hat{C}}_{j_{1}}$, denoted as ${\hat{C}}_{j_{x_{1}}}$ and ${\hat{C}}_{j_{x_{2}}}$, ${}^{j_{x_{1}}}{\hat{C}}_{i_{x_{1}}}$ and a ${}^{j_{x_{2}}}{\hat{C}}_{i_{x_{2}}}$ should be derived from ${\{}^{j_{x_{1}}}\hat{C}\}$ and ${\{}^{j_{x_{2}}}\hat{C}\}$, respectively, satisfying
  $$▵\;{}^{j_{1}}{\hat{C}}_{i_{1}}{}^{j_{x_{1}}}{\hat{C}}_{i_{x_{1}}}{}^{j_{x_{2}}}{\hat{C}}_{i_{x_{2}}}\approx ▵\;{\hat{C}}_{j_{1}}{\hat{C}}_{j_{x_{1}}}{\hat{C}}_{j_{x_{2}}}$$
  and
  (11) $$\begin{array}{cc} (∥\vec{{}^{j_{1}}{\hat{C}}_{i_{1}}{\;}^{j_{x_{1}}}{\hat{C}}_{i_{x_{1}}}}∥ & -∥\vec{{\hat{C}}_{j_{1}}\;{\hat{C}}_{j_{x_{1}}}}{∥)}^{2}<{T\;}^{2}\\ (∥\vec{{}^{j_{1}}{\hat{C}}_{i_{1}}{\;}^{j_{x_{2}}}{\hat{C}}_{i_{x_{2}}}}∥ & -∥\vec{{\hat{C}}_{j_{1}}\;{\hat{C}}_{j_{x_{2}}}}{∥)}^{2}<{T\;}^{2}\\ (∥\vec{{}^{j_{x_{1}}}{\hat{C}}_{i_{x_{1}}}{\;}^{j_{x_{2}}}{\hat{C}}_{i_{x_{2}}}}∥ & -∥\vec{{\hat{C}}_{j_{x_{1}}}\;{\hat{C}}_{j_{x_{2}}}}{∥)}^{2}<{T\;}^{2}. \end{array}$$

The conditions are applied one by one. Under the first two conditions, more than 80% of unqualified candidates can be filtered out in our experiments. Under the last condition, at most one candidate can be left in each set. Finally, several cluster correspondences between $\left\{\hat{C}\right\}$ and {C} are obtained. The transformation T between the local and global ClusterMaps is then calculated by

(12) $$T=arg\;\underset{T}{min}\;\frac{1}{2}\;\sum^{m_{suc}}{∥O_{i}-T{\hat{O}}_{j}∥}^{2},$$

where $O_{i}$ and ${\hat{O}}_{j}$ are the virtual center points of the cluster mentioned in Section 3.2, and $m_{suc}$ is the number of remaining matching pairs after applying RANSAC selection. The RANSAC algorithm is used to eliminate the effects of possible mismatches and ultimately obtain more reliable matching results.

All candidates in each ${\{}^{j}\hat{C}\}$ are transversed. Thus, the algorithm of geometric verification may reach a poor complexity $O(n^{N_{l}})$, where *n* is the maximum size of ${\{}^{j}\hat{C}\}$. However, because the size of each ${\{}^{j}\hat{C}\}$ is generally ~2–15, the value of *n* is of small order of magnitude. By sequentially applying the three geometric conditions, a considerable number of candidate outliers are filtered out. The results show that not much time is spent on geometry verification. The time consumption of the entire relocalization process is evaluated in Section 5.

## 5. Experiments

**Figure 8:**
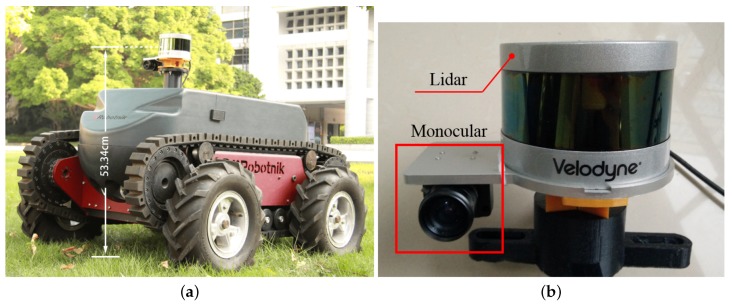
Our experimental platform: (**a**) Robotnik Guardian equipped with a monocular camera and a 3D LIDAR, and (**b**) sensor module including a Pointgrey FMVU-03MTM-CS and a Velodyne VLP-16.

**Figure 9:**
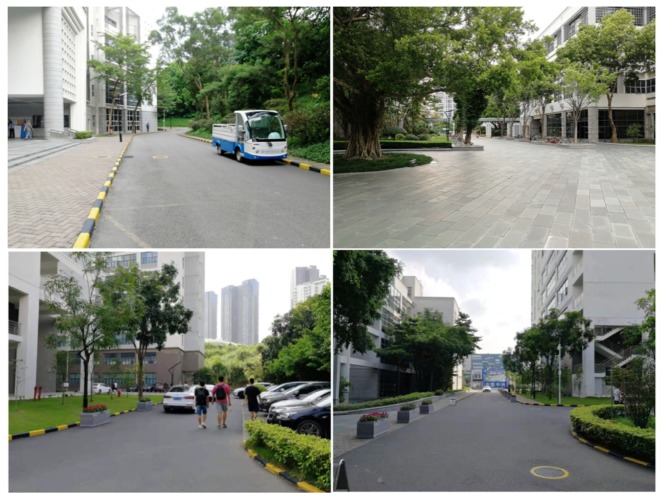
Photos of our campus environment.

In this section, several experiments are performed to illustrate the effectiveness of the proposed algorithms, i.e., ClusterMap building and ClusterMap-based relocalization. The KITTI dataset [34] for autonomous driving is used as a benchmark in performance evaluation. All experiments are tested on a laptop computer with Intel i7-4900MQ CPU@2.80 GHz and 6 GB memory. The proposed algorithms are evaluated in our campus with our unmanned vehicle platform, whose sensor configuration is illustrated in Figure 8. The sensor module is equipped 0.7 m above the ground surface. The LIDAR is operated with 10 Hz measurement speed, and the vehicle is moving at a maximum speed of 2 m/s. Only the information from the camera and LIDAR is used to complete the motion estimation. Figure 9 illustrates some snapshots of our experimental environment. Our campus is a part of Shenzhen’s urban environment, which has moving pedestrians and parking cars. All the algorithms are implemented with C++.

### 5.1. Evaluation on KITTI Data Set

**Figure 10:**
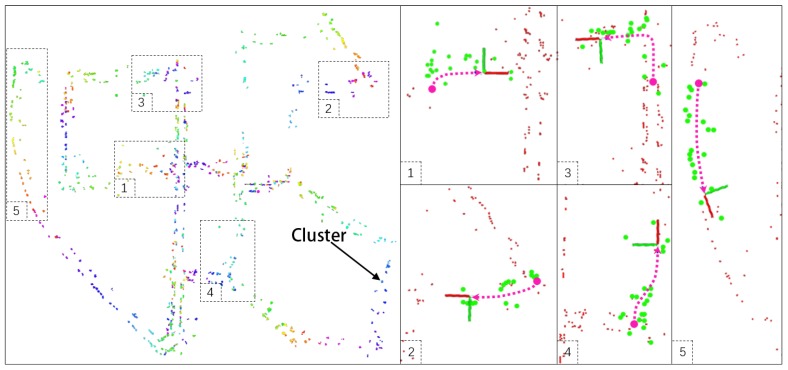
Experimental results of relocalization on KITTI dataset.

To verify the proposed map format and the relocalization ability, an evaluation experiment is first performed on KITTI dataset. In this experiment, we utilize ORB-SLAM to estimate the robotic trajectory; on the basis of which the ClusterMap of KITTI sequence 00 is built as illustrated in Figure 10. The figure on the left shows all 3D clusters in the KITTI 00 scene, whereas the right one shows the results of the relocalization tests. Five tests are performed where the locations are randomly selected. In the right subfigures of Figure 10, red points denote clusters in the global ClusterMap, green ones indicate clusters of the local map, and each pink dotted line indicates the vehicle path to establish the local map. The places in the global map are randomly selected to evaluate the relocalization performance. The perceived data at these local places are obtained from the original dataset. As shown in Figure 10, the vehicle relocalizes successfully at all places. However, the relocalization on KITTI is ideal because the locally perceived data for the local map is the same as that for building the global ClusterMap, i.e., illumination, objects, and other conditions are exactly the same.

### 5.2. Evaluation with Our Experimental Vehicle

**Figure 11:**
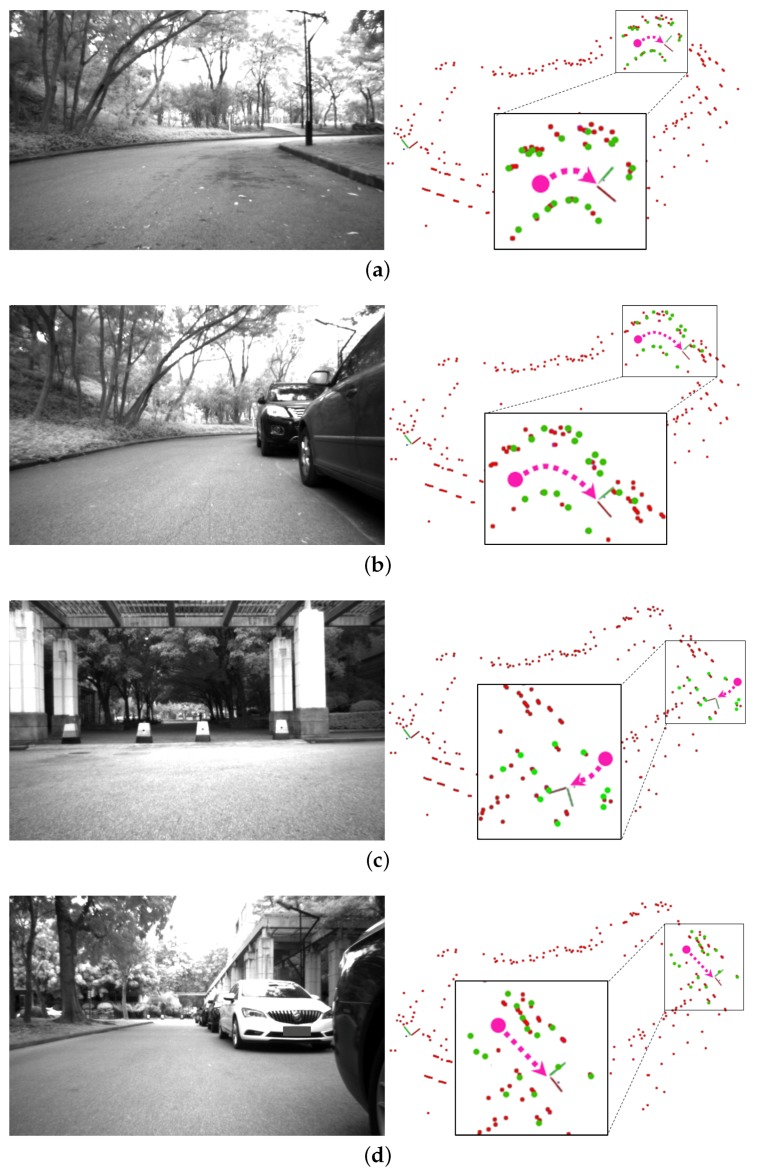
Experiments of relocalization in our campus. (**a**,**b**) The results at the same location but at different times with a three-month interval; (**c**,**d**) the results at the same location but from different entry directions.

To verify the proposed approach, we perform an experiment in our campus, as shown in Figure 11. We use the experimental platform shown in Figure 8, which is equipped with VLP-16 of low density. As shown in Figure 9, the campus is a typical urban environment with various natural objects, e.g., trees, shrubs, concrete columns, parking cars, and walking pedestrians. The vehicle shown in Figure 8a is driven manually in the campus. The global ClusterMap is initially built by using the SLAM method given in Section 3.1. Then, the vehicle is manually driven after three months to verify the relocalization algorithm on the basis of the built ClusterMap. Within three months, the environmental appearance presents significant changes, e.g., parked vehicles disappear, and trees grow. In accordance with the experimental results, the relocalization performs well in our campus. The experimental video can be found in the supplementary materials.

Two difficult cases are demonstrated for the relocalization, as shown in Figure 11. In each part, the figure on the left is a monocular image, whereas the right one shows the relocalization result. In the first case, as shown in Figure 11a,b, the parked vehicles disappear, and the shrubs grow after three months; the visual appearance changes significantly and leads to difficulty in relocalization. The results show that the relocalization is reliable despite large occlusions in scenes. However, long paths are required to collect additional local clusters. In the second case, as shown in Figure 11c,d, the vehicle enters the same place but from different directions. The perceived visual appearances are totally different due to the different directions and occlusion situation. Therefore, the relocalization is challenging if existing visual approaches are used. Nevertheless, the spatial relationships among long-term static objects are stable, even when some objects are occluded or observed from different directions. The relationships are considered in the proposed approach. As such, the relocalization in the two kinds of difficult cases is completed successfully.

### 5.3. Parameters Evaluation

**Figure 12:**
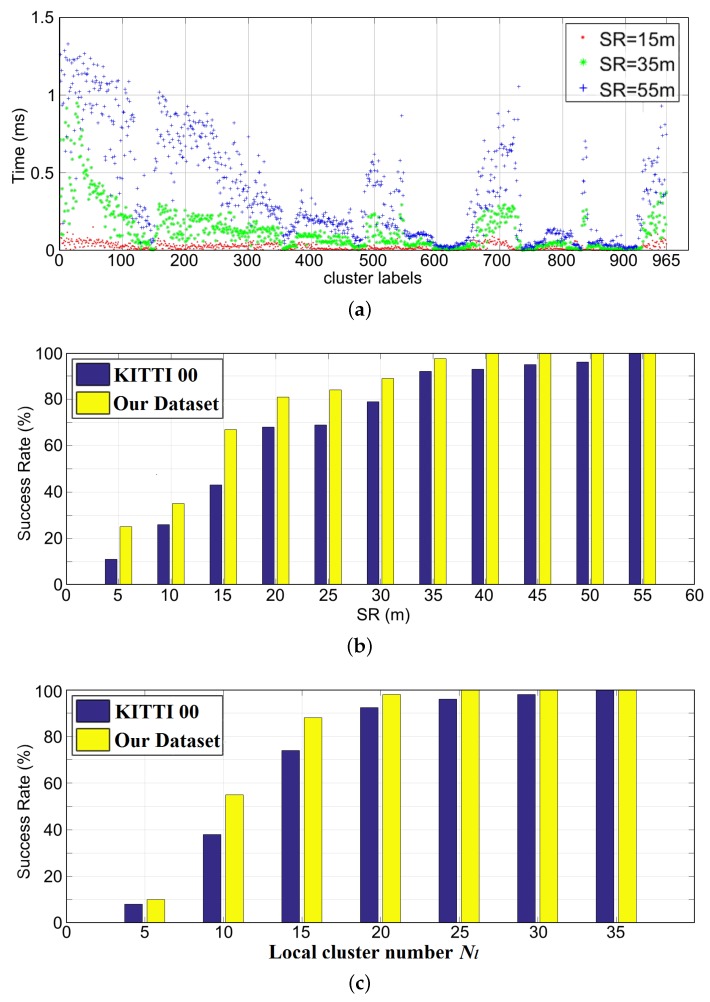
Performance evaluation of the algorithm parameters SR and cluster number $N_{l}$ in local ClusterMap. (**a**) Computation time of establishing each location descriptor under different SR values; (**b**,**c**) visualization of the success rate of relocalization versus different values of SR and $N_{l}$.

As stated in Section 3.3, the parameter of search radius SR plays an important role in the performance of cluster descriptors. Figure 12a shows the numerical statistics of the computation time for establishing each descriptor under different values of SR on KITTI sequence 00 and the dataset from our campus environment. The built ClusterMap contains 965 clusters. Thus, 965 location descriptors are established. The cluster number in the local ClusterMap also affects the performance. Therefore, the number of local clusters is set to 20 in evaluating the parameter SR. Figure 12b shows the histogram chart of the relocalization success rate versus SRs. In the experiment, for each different SR condition, 100 tests are performed on the ClusterMaps built from the KITTI sequence 00 (Figure 10) and the dataset from our campus environment (Figure 3). The testing places are randomly selected. The global ClusterMap building and relocalization experiments are performed on the same KITTI dataset. However, in terms of our dataset, the situation is different. All the datasets used for relocalization are collected after three months. Thus, they are different from those used for building the global map.

As shown in Figure 12a,b, the larger the value of SR, the more time it consumes, but the higher the success rate of the relocalization. To evaluate the factor of cluster number in the local ClusterMap further, we fix the value of SR to 35 m. The same method is used to obtain the relocalization success rates with different cluster numbers in the local ClusterMap. Figure 12c illustrates the experimental result. Large cluster numbers in local maps increase success rates. As shown in Figure 12c, when the value of SR is set to 35 m with the number of local clusters as 20, the relocalization success rate reaches more than 92% and 97% in the KITTI sequence 00 and our campus datasets, respectively.

**Figure 13:**
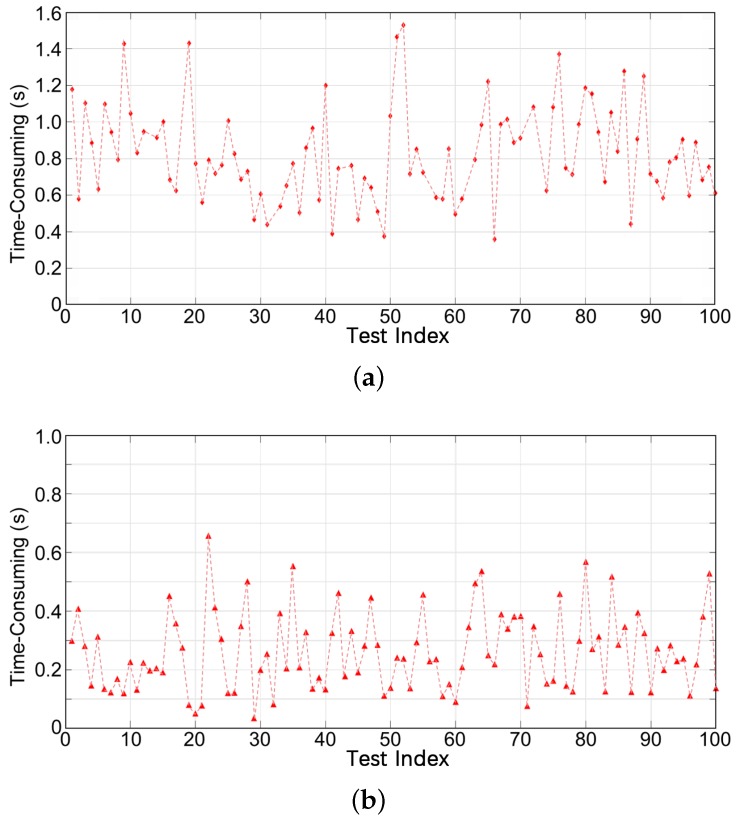
Computation time evaluation of the match process on (**a**) KITTI sequence 00 and (**b**) our campus dataset.

With the same parameters, another 100 tests are performed to evaluate the computation time of the entire relocalization. The relocalization includes creating all descriptors for the local ClusterMap, completing the descriptor matching, and implementing geometric verification. Figure 13 illustrates the experimental results, where the horizontal axis is the test index. The duration of the entire process is short, and relocalization is achieved quickly. Because the GPS information is unstable in urban environments especially in the places with many buildings, the ground truth is unable to provide for the evaluation in our dataset.Therefore, we define the reprojection error as

(13) $$e=\sqrt{\frac{\sum^{m_{suc}}{∥O_{i}-T{\hat{O}}_{j}∥}^{2}}{m_{suc}}}.$$

**Figure 14:**
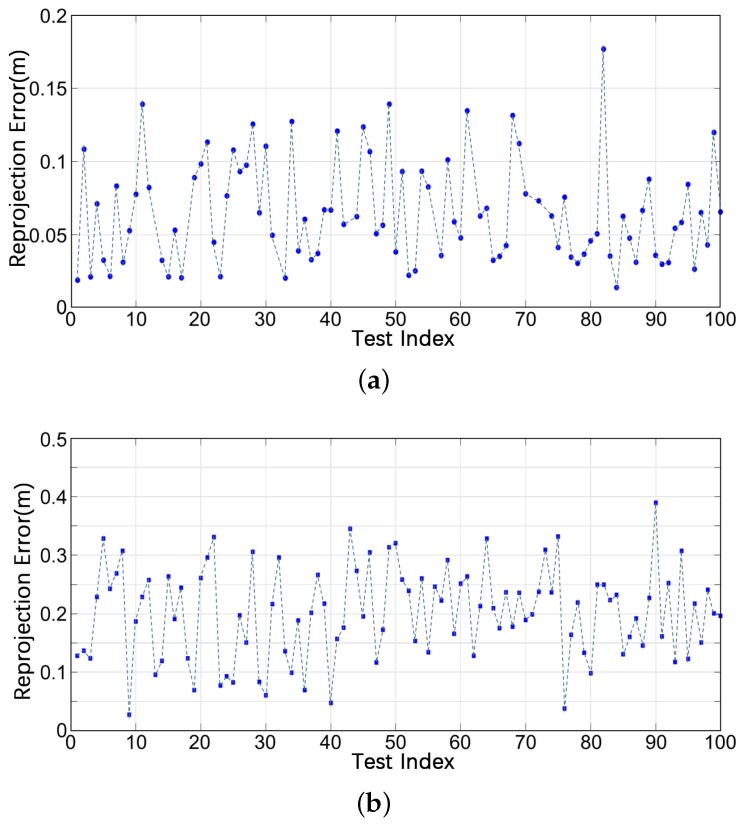
Reprojection error of the entire process on the (**a**) KITTI sequence 00 and (**b**) our campus dataset.

Figure 14 illustrates the reprojection errors of different tests. The reprojection errors performed on our experimental platform in our campus are approximately ~3–5 times the errors of KITTI sequence 00. Two reasons can explain this result. First, the LIDAR equipped on our platform is a Velodyne VLP-16, whereas the one in KITTI is a Velodyne HDL-64E. Second, our own datasets for evaluation are recorded using our experimental platform at different times with significant time intervals; many changes occur in environmental appearances and vehicle trajectories. The environmental changes cause a large difference between local and global ClusterMaps. Even for the same object, the virtual center point $O_{i}$ of its point cluster obtained by the simple clustering algorithm is inconsistent in different maps. Nevertheless, on the basis of the experimental results, the relocalization success rate still reaches a high level. The ClusterMap-based relocalization method exhibits strong robustness to noise and long-term environmental changes.

**Figure 15:**
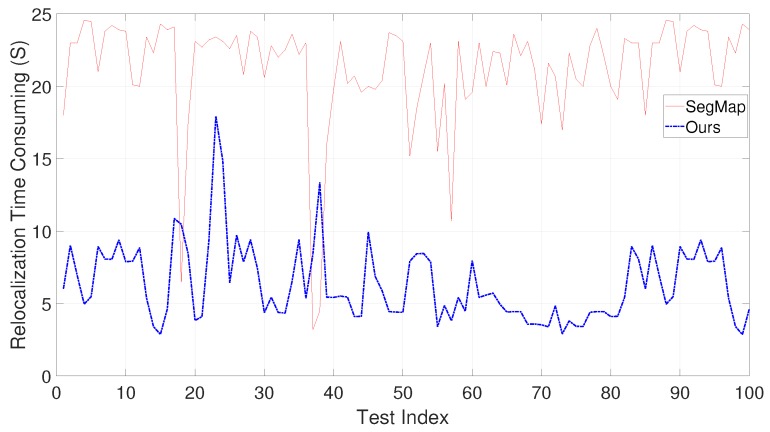
Comparison on the computation time of the entire process on KITTI sequence 00 between SegMap and ours.

**Figure 16:**
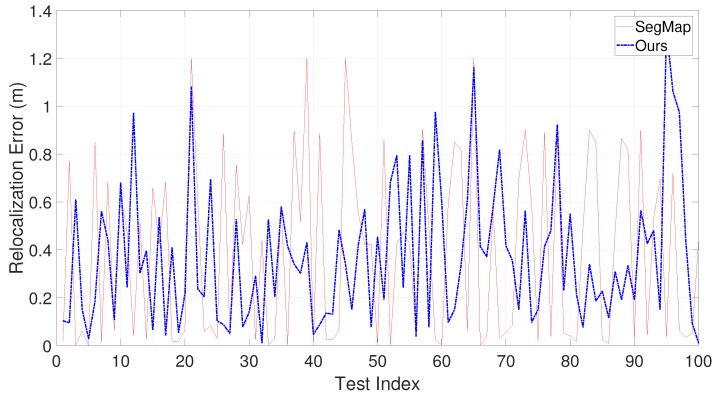
Comparison on the relocalization error on KITTI sequence 00 between SegMap and ours.

To further illustrate the performance, experiments are also performed to compare with the recently published work SegMap [28]. SegMap utilizes 3D LIDAR information only and can achieve both localization and relocalization. We perform 100 tests by randomly selecting the relocalization positions. Because KITTI provides ground truth for evaluation, both the time consumption and relocalization error are compared. Figure 15 shows the computation time’s comparison between SegMap and the proposed approach. Note that the computation time of our approach in Figure 15 contains the entire time for local map construction and map matching; therefore, the time is longer than that in Figure 13. Figure 16 shows the relocalization error of SegMap and the proposed approach. From Figure 15 and Figure 16, it is seen that although the relocalization accuracy of our approach is similar or a little bit worse, the computation time is much lower than that of SegMap. The poorer accuracy may derive from the simple cluster extraction in our approach. It will be our future work to improve cluster extraction.

### 5.4. Discussion

From the experiments, it is seen that the proposed approach need to travel for a certain distance to collect enough local clusters. The limitation derives from the compact character of ClusterMap, because ClusterMap is composed with only salient clusters detected from the primary point clouds. However, the relocalization is achieved quickly, ~3–10 s based on our experiments, and it will not be a problem in practical applications. Other solutions, like the SegMatch-based approach [27], require high-density point clouds for describing keypoints or segments. In addition, the feature descriptors are susceptible to environmental changes in long-term applications, in which shrubs or trees may change greatly, thereby causing a large change in the feature descriptors of the same object at different times. In contrast, our approach can address the problems. Therefore, the proposed approach can be applied in the low-speed fields with autonomous logistic cars, surveillance robots, or sanitation vehicles, and so on. These applications have strong requirements of low-cost and high performance.

## 6. Conclusions

The study proposes a novel representation of environmental maps called ClusterMap. The ClusterMap is composed of point cloud clusters, each of which is associated with a descriptor describing the location relationship with neighboring clusters. The new kind of map directly represents the distribution of long-term static objects in complex environments. To build a ClusterMap that is consistent with a real operating environment without scale problems, we utilize the multisensor fusion approach that combines visual features and LIDAR’s point clouds. This approach can compensate for the shortcomings of different sensors and obtain low-scale drift localization and mapping results. Furthermore, a novel cluster descriptor and its matching algorithm are developed for finding cluster correspondences among different ClusterMaps. On the basis of the ClusterMap building and cluster descriptor, a robust relocalization algorithm is developed. Finally, several experiments are performed on well-known datasets and on our own experimental vehicle in our campus environment to illustrate the performance of the proposed algorithms.

## Supplementary Materials

The following are available online at https://www.mdpi.com/1424-8220/19/19/4252/s1, Video S1: Experiments of ClusterMap Building and Relocalization in Urban Environments for Unmanned Vehicles.

## Abbreviations

The following abbreviations are used in this manuscript.

SLAM	Simultaneous Localization and Mapping
PFHs	Point Feature Histograms
FPFHs	Fast Point Feature Histograms

## References

[1] Cadena C., Carlone L., Carrillo H., Latif Y., Scaramuzza D., Neira J., Reid I.D., Leonard J.J. (2016). Past, Present, and Future of Simultaneous Localization and Mapping: Toward the Robust-Perception Age. IEEE Trans. Robot..

[2] Zhang J., Singh S. (2018). Laser–visual–inertial odometry and mapping with high robustness and low drift. J. Field Robot..

[3] Wang H., Guo D., Liang X., Chen W., Hu G., Leang K.K. (2017). Adaptive vision-based leader-follower formation control of mobile robots. IEEE Trans. Ind. Electron..

[4] Lin L.S., Yang Y.J., Cheng H., Chen X.C. (2019). Autonomous Vision-Based Aerial Grasping for Rotorcraft Unmanned Aerial Vehicles. Sensors.

[5] Schauwecker K., Zell A. Robust and efficient volumetric occupancy mapping with an application to stereo vision. Proceedings of the IEEE International Conference on Robotics and Automation.

[6] Bogoslavskyi I., Stachniss C. (2017). Efficient online segmentation for sparse 3d laser scans. Photogramm. Remote Sens. Geoinf. Sci..

[7] Lynen S., Achtelik M.W., Weiss S., Chli M., Siegwart R. A robust and modular multisensor fusion approach applied to mav navigation. Proceedings of the Intelligent Robots and Systems (IROS).

[8] Wan G., Yang X., Cai R., Li H., Wang H., Song S. (2017). Robust and Precise Vehicle Localization based on Multi-sensor Fusion in Diverse City Scenes. arXiv.

[9] Mur-Artal R., Montiel J.M.M., Tardos J.D. (2015). ORB-SLAM: A versatile and accurate monocular SLAM system. IEEE Trans. Robot..

[10] Engel J., Schöps T., Cremers D. LSD-SLAM: Large-scale direct monocular SLAM. Proceedings of the European Conference on Computer Vision.

[11] Engel J., Koltun V., Cremers D. (2018). Direct Sparse Odometry. IEEE Trans. Pattern Anal. Mach. Intell..

[12] Mur-Artal R., Tardós J.D. (2017). Orb-slam2: An open-source slam system for monocular, stereo, and rgb-d cameras. IEEE Trans. Robot..

[13] Grisetti G., Stachniss C., Burgard W. (2007). Improved techniques for grid mapping with rao-blackwellized particle filters. IEEE Trans. Robot..

[14] Hess W., Kohler D., Rapp H., Andor D. Real-time loop closure in 2D LIDAR SLAM. Proceedings of the Robotics and Automation (ICRA).

[15] Zhang J., Singh S. (2017). Low-drift and real-time lidar odometry and mapping. Auton. Robot..

[16] Pfrunder A., Borges P.V., Romero A.R., Catt G., Elfes A. Real-time autonomous ground vehicle navigation in heterogeneous environments using a 3D LiDAR. Proceedings of the Intelligent Robots and Systems (IROS).

[17] Opromolla R., Fasano G., Rufino G., Grassi M., Savvaris A. LIDAR-inertial integration for UAV localization and mapping in complex environments. Proceedings of the Unmanned Aircraft Systems (ICUAS).

[18] Brenneke C., Wulf O., Wagner B. Using 3d laser range data for slam in outdoor environments. Proceedings of the Intelligent Robots and Systems.

[19] Wang L., Zhang Y., Wang J. (2017). Map-based localization method for autonomous vehicles using 3D-LIDAR. IFAC-Papersonline.

[20] Zhang J., Kaess M., Singh S. (2017). A real-time method for depth enhanced visual odometry. Auton. Robot..

[21] Lenac K., Kitanov A., Cupec R., Petrović I. (2017). Fast planar surface 3D SLAM using LIDAR. Robot. Auton. Syst..

[22] Zhu Z., Yang S., Dai H., Li F. Loop Detection and Correction of 3D Laser-Based SLAM with Visual Information. Proceedings of the 31st International Conference on Computer Animation and Social Agents.

[23] Chen H., Huang H., Qin Y., Liu Y. (2019). Vision and Laser Fused SLAM in Indoor Environments with Multi-Robot System. Assem. Autom..

[24] Karami E., Prasad S., Shehata M.S. (2017). Image Matching Using SIFT, SURF, BRIEF and ORB: Performance Comparison for Distorted Images. arXiv.

[25] Bosse M., Zlot R. Place recognition using keypoint voting in large 3D lidar datasets. Proceedings of the Robotics and Automation (ICRA).

[26] Gawel A., Cieslewski T., Dubé R., Bosse M., Siegwart R., Nieto J. Structure-based vision-laser matching. Proceedings of the 2016 IEEE/RSJ International Intelligent Robots and Systems (IROS).

[27] Dubé R., Dugas D., Stumm E., Nieto J., Siegwart R., Cadena C. (2016). Segmatch: Segment based loop-closure for 3d point clouds. arXiv.

[28] Dubé R., Cramariuc A., Dugas D., Nieto J., Siegwart R., Cadena C. SegMap: 3D Segment Mapping using Data-Driven Descriptors. Proceedings of the Robotics: Science and Systems (RSS).

[29] Finman R., Paull L., Leonard J.J. Toward object-based place recognition in dense rgb-d maps. Proceedings of the ICRA Workshop Visual Place Recognition in Changing Environments.

[30] Rusu R.B., Blodow N., Marton Z.C., Beetz M. Aligning point cloud views using persistent feature histograms. Proceedings of the Intelligent Robots and Systems.

[31] Rusu R.B., Blodow N., Beetz M. Fast point feature histograms (FPFH) for 3D registration. Proceedings of the Robotics and Automation.

[32] Scaramuzza D., Fraundorfer F. (2011). Visual odometry [tutorial]. IEEE Robot. Autom. Mag..

[33] Dhall A., Chelani K., Radhakrishnan V., Krishna K.M. (2017). LiDAR-Camera Calibration using 3D–3D Point correspondences. arXiv.

[34] Geiger A., Lenz P., Stiller C., Urtasun R. (2013). Vision meets Robotics: The KITTI Dataset. Int. J. Robot. Res..

